# Evaluating the bias of circRNA predictions from total RNA-Seq data

**DOI:** 10.18632/oncotarget.22972

**Published:** 2017-12-06

**Authors:** Jinzeng Wang, Kang Liu, Ya Liu, Qi Lv, Fan Zhang, Haiyun Wang

**Affiliations:** ^1^ School of Life Sciences and Technology, Tongji University, Shanghai 200092, China; ^2^ National Research Center for Translational Medicine (Shanghai), Rui Jin Hospital, Shanghai Jiao Tong University School of Medicine, Shanghai 200025, China; ^3^ Clinical Translational Research Center, Shanghai Pulmonary Hospital, Tongji University School of Medicine, Shanghai 200433, China

**Keywords:** circular RNA, circRNA predictions, total RNA-Seq, CIRI, KNIFE

## Abstract

CircRNAs are a group of endogenous noncoding RNAs. The quickly developing high throughput RNA sequencing technologies along with novel bioinformatics approaches have enabled researchers to systematically identify circRNAs and their biological functions in cells. Deep sequencing of rRNA-depleted RNAs treated with RNase R, which digests linear RNAs and leaves circRNAs enriched, is an efficient way to identify circRNAs. However, very few of RNase R treated data are at hand but a large amount of total RNA-Seq data with no sequencing costs is available, for circRNA predictions. In this study, we systematically investigated the prediction bias from total RNA-Seq data as well as the influence of sequencing depth, sequencing quality and single-end or paired-end sequencing strategy on the predictions. We also identified circRNA properties that may contribute to the improved prediction performance. Our analysis shows that circRNA predictions from total RNA-Seq data gain ∼50% true positive. Sequencing error dramatically worsens the predictions, rather than single-end sequencing strategy or low sequencing depth. However, false positive can be carefully controlled by using data with good quality and narrowing down circRNAs guided by their properties.

## INTRODUCTION

Circular RNAs (circRNAs) have been discovered as a novel group of endogenous noncoding RNA. Differing from linear RNAs, circRNAs lack 5′ cap and 3′ poly(A) tail and form covalently closed loop structures with circularization of exon, intron or intergenic regions, which makes them more conserved and stable [1, 2]. CircRNAs are resistant to RNase R, an RNA exonuclease that preferentially degrades linear RNAs [3]. CircRNAs may act as microRNA sponges, interact with RNA binding proteins, regulate alternative splicing or transcription and translate into the protein of parental genes [4, 5]. More importantly, circRNAs may have great potential roles in biological development and disease initiation or progression, and can function as new clinical diagnostic and prognostic biomarkers [6–12]. For instance, expression levels of circRNAs are significantly increased in cancer serum compared to those in normal serum, so circRNAs can serve as a promising biomarker for cancer diagnosis [9]. Therefore, circRNAs have been back to forefront of the RNA field and attain much more attention from basic research to clinical application.

CircRNAs were first found in a viroid as early as 1970s [13], unfortunately such molecules were long considered to be aberrant RNA splicing byproducts or a few of specific pathogens due to their low expression abundance [14, 15]. Promisingly, the quickly developing high throughput RNA deep sequencing technologies along with bioinformatics approaches [6, 16–21] have enabled researchers to systematically identify circRNAs and their biological functions in cells. Ideally, paired-end deep sequencing of rRNA-depleted RNAs treated with RNase R, which can digest linear RNAs while leave circRNAs unaffected, is an efficient way to identify circRNAs. However, very few of such ‘perfect’ data are at hand but a great deal of ‘imperfect’ total RNA-Seq data with no RNase R treatment, even with single-end sequencing, low sequencing quality or poor sequencing depth are available for circRNAs predictions.

Presently, there are few studies investigate the prediction bias, as well as the factors that contribute to the improved predictions, from total RNA-Seq data. In this study, we used a de novo circRNA prediction algorithms CIRI [16] and KNIFE [17] to predict circRNAs from total RNA-Seq data, then evaluated prediction performance using RNase R treated sample. We further generated three types of simulated data based on total RNA-Seq data, which respectively simulated the imperfect RNA-Seq data with single-end sequencing, gradually decreased sequence quality or sequencing depth. The prediction performance was also evaluated on these imperfect data. Finally, we investigated the circRNAs properties that can contribute to the efficient prediction of true circRNAs.

## RESULTS

### Predicting the circRNAs from total RNA-Seq data

Hansen *et al.* employed Hs68 fibroblast to compare the performance of five circRNA prediction algorithms [22]. Here we used the same samples to investigate the bias of circRNA predictions from total RNA-Seq data. Two replicates of total RNA-Seq data with 100-bp paired-end reads from Hs68 (SRR444655, SRR444975) were used in this study. FastQC analysis (http://www.bioinformatics.babraham.ac.uk/projects/fastqc/) showed the good quality of four single-end RNA-Seq data, including SRR444655_1, SRR444655_2, SRR444975_1 and SRR444975_2, except for a declined quality at the 3′ termini of the reads (Supplementary Figure 1A, 1B). This decline was more obvious for the second single-end data SRR444655_2 (Supplementary Figure 1A, right panel). We applied the recently published circRNA prediction algorithms CIRI (CIRI1.2 and CIRI2.0) and KNIFE to predict novel circRNAs from total RNA-Seq data, since CIRI algorithm have showed the advantage over the other two algorithms, segemehl and find_circ [22]. As for KNIFE algorithm, unlike previous algorithms that were lack of rigorous statistical testing, it used a statistical approach to decrease false positive identification [17]. To distinguish true circRNAs from false ones, two replicates of RNase R treated RNA-Seq data in Hs68 (SRR444974, SRR445016) were used as a validation. The circRNAs with the reads enriched in RNase R treated samples were reported as true positives based on enrichment score (See methods). FastQC analysis also showed the good quality for SRR444974 and SRR445016 except for a poor quality at the 3′ termini of the reads (Supplementary Figure 1C, 1D).

**Figure 1:**
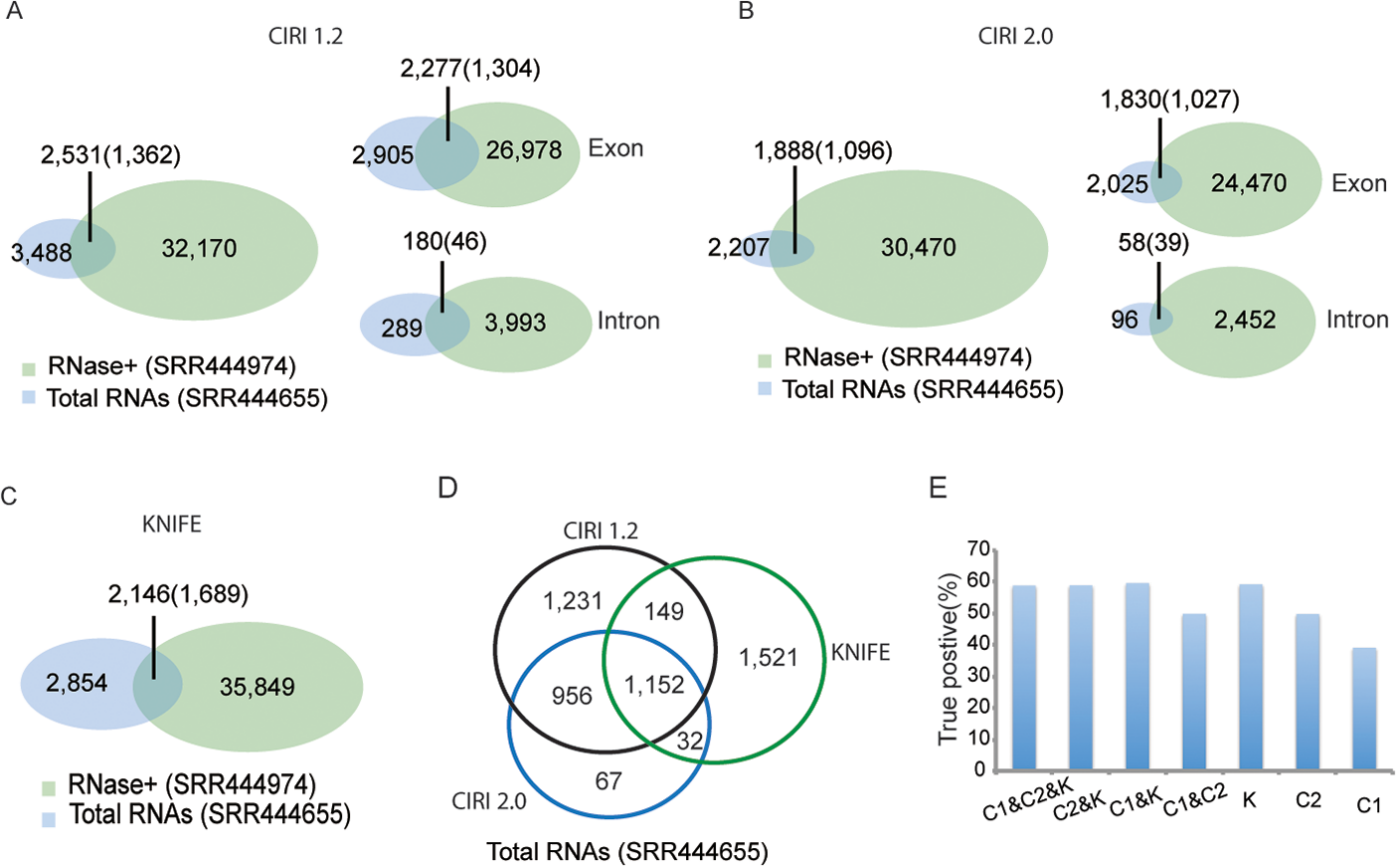
Prediction performance of the total RNA-Seq data SRR444655 using three algorithms Prediction performance of the total RNA-Seq data using CIRI1.2 (**A**), CIRI2.0 (**B**) or KNIFE (**C**). Most of predicted circRNAs and true positives were generated by exon circularization. An overlap of 1,152 circRNAs was observed between all algorithms (**D**). Prediction performance was improved when using combining more than 2 algorithms. C1, C2 and K represent CIRI1.2, CIRI2.0 and KNIFE, respectively (**E**).

From total RNA-Seq data SRR444655, the algorithms respectively predicted 3,488 (CIRI1.2), 2,207 (CIRI2.0), and 2,854 (KNIFE) circRNAs with at least two reads spanning the splicing sites (Figure 1). As expected, the number of circRNAs predicted by using RNase R treated data SRR444974, with 32,170 for CIRI1.2, 30,470 for CIRI2.0, and 35,849 for KNIFE, was much greater than one using total RNAs samples (Figure 1). CIRI1.2 predicted 2,531 circRNAs that were both found by RNase R treated samples and total RNA samples. Among them 1,362 circRNAs were enriched in RNase R treated sample (enrichment score E > 0) and thus defined as true circRNAs. Comparing with 3,488 circRNAs predicted from total RNA-Seq, the true positive rate of CIRI1.2 is 39.05%. And CIRI2.0 and KNIFE algorithms respectively found 1,096 (49.71%) and 1,689 (59.18%) true circRNAs. The complete list of true circRNAs and false circRNAs was shown in Supplementary Tables 1 and 2. Most of predicted circRNAs and true positives were generated by exon circularization. Intron circularization was also identified. KNIFE algorithm only identified the circRNAs from exons. Moreover, most of circRNAs were generated from protein coding genes (Supplementary Table 3). CircRNAs predicted only by one approach were more likely to be false positive in general [22], and a recent evaluation based on 11 circRNA prediction tools also showed that no single approach dominated on all of the metrics of performance [23]. So several algorithms should be combined to achieve reliable predictions. In our analysis, of the predicted circRNAs using three algorithms, an overlap of 1,152 circRNAs was observed between all algorithms (Figure 1D). Prediction performance was improved when combining more than 2 algorithms (Figure 1E).

The results analysed using total RNA-Seq data SRR444975 and RNase R treated RNA-Seq data SRR445016 by CIRI2.0 were shown in Supplementary Figure 2.

### Evaluating the bias of predicted circRNAs from the imperfect total RNA-Seq data

The above analysis suggested that it was feasible to predict the circRNAs from total RNA-Seq data, though prediction should be cautious due to ∼50% false positive. Therefore, a large amount of total RNA-Seq data in the public databases can be for the effective use of novel circRNA discovery. However, the public total RNA-Seq data may not be as perfect as SRR444655 and SRR444975 due to single-end sequencing, poor sequence quality or low sequencing depth.

**Figure 2:**
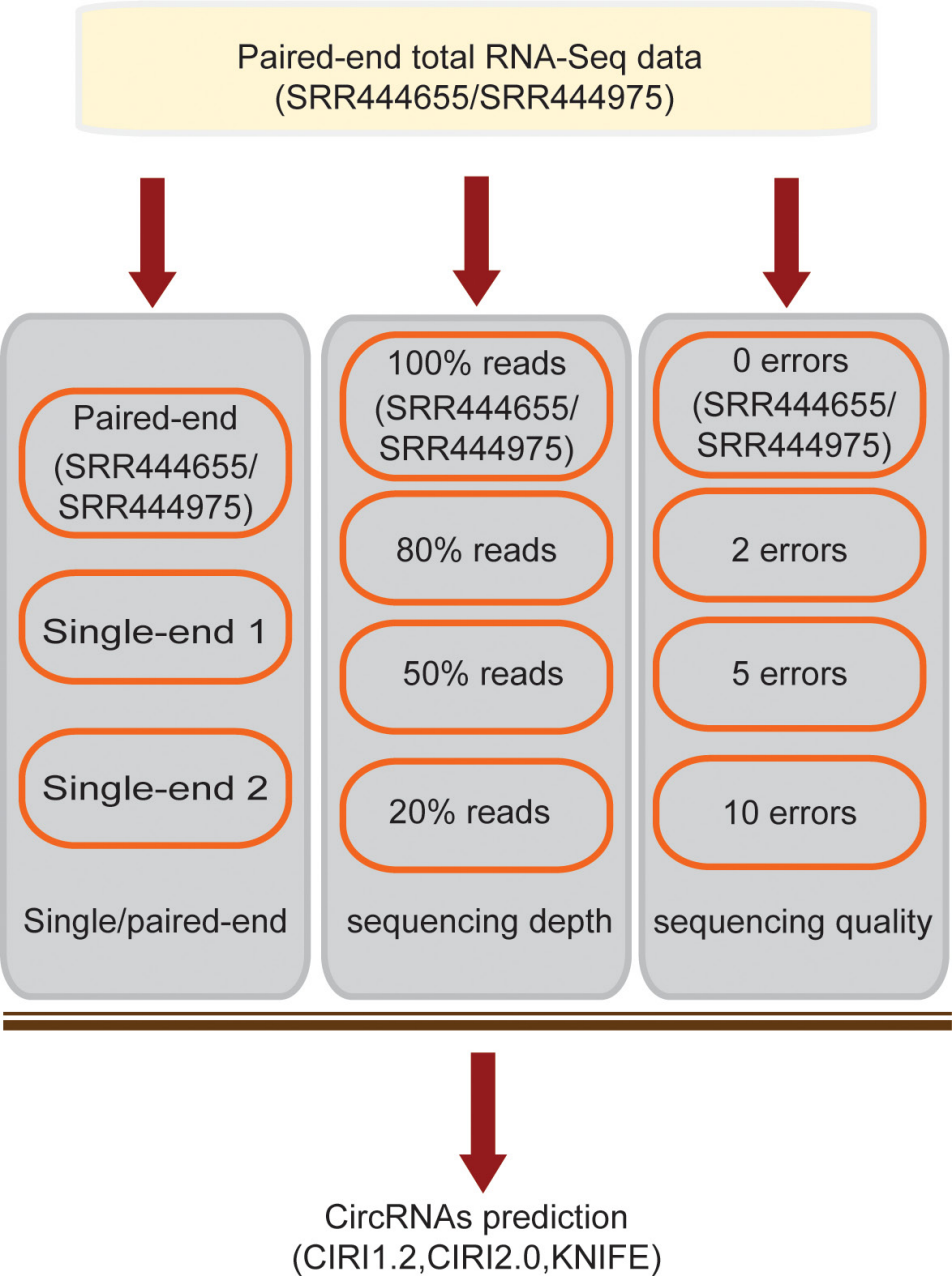
Pipeline for generating the simulated imperfect RNA-Seq data

To systematically evaluate the prediction bias of circRNAs from the imperfect total RNA-Seq data, we generated three types of simulations (Figure 2): (i) Only single-end reads extracted from SRR444655/SRR444975, simulating single-end sequencing strategy, were used for prediction; (ii) Reads randomly sampled from SRR444655/SRR444975 with a different sampling rate, simulating the gradually decreased sequencing depth, were used for prediction and (iii) Reads from SRR444655/SRR444975 with the bases of each read being replaced by other false bases, simulating decreased sequencing quality, were used for prediction.

**Figure 3:**
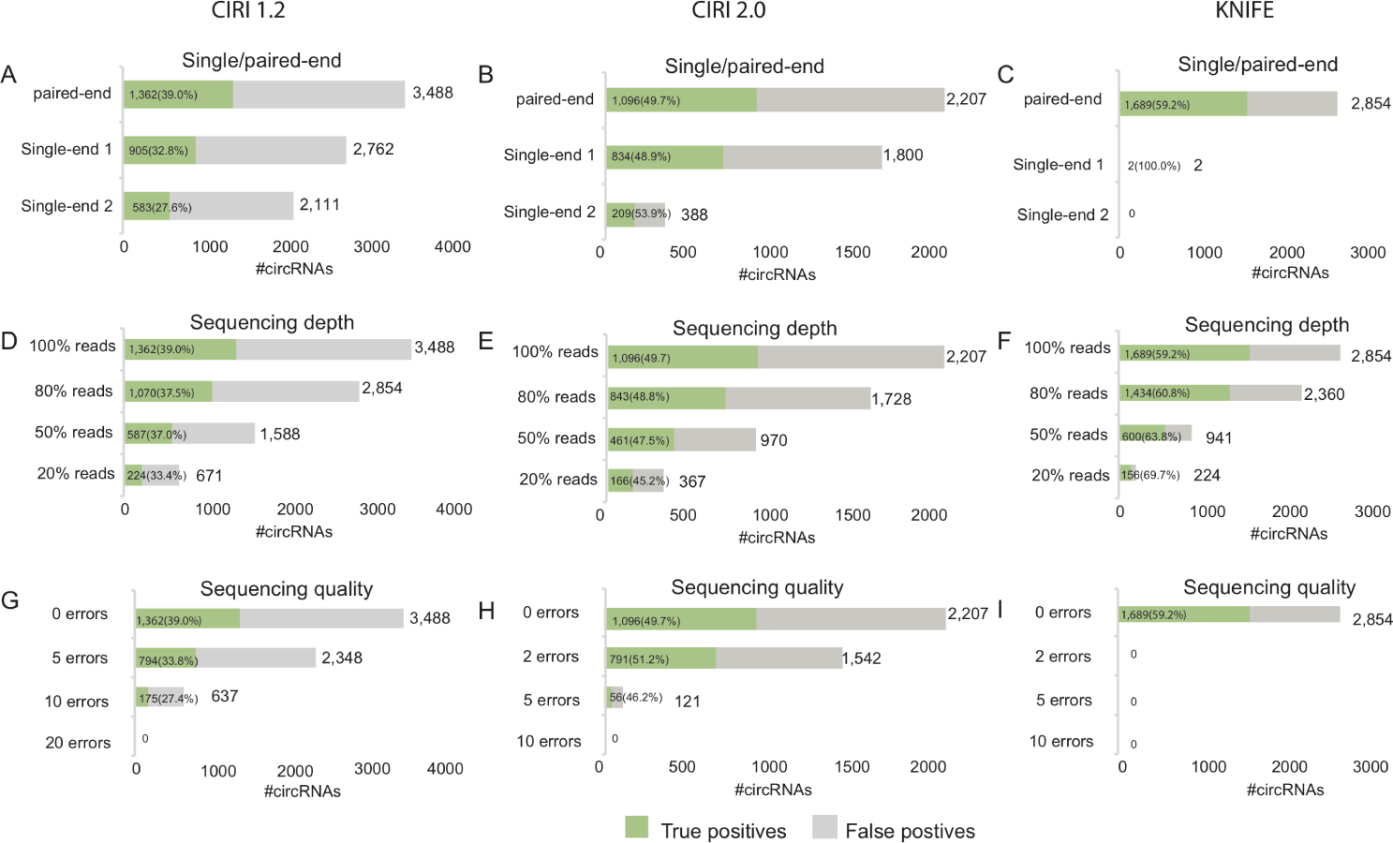
Prediction performance of the simulated data using three algorithms Prediction performance of the single-end sequencing data using CIRI1.2 (**A**), CIRI2.0 (**B**) or KNIFE (**C**). Prediction performance of the data with gradually decreased sequencing depth using CIRI1.2 (**D**), CIRI2.0 (**E**) or KNIFE (**F**). Prediction performance of the data with gradually increased sequencing error using CIRI1.2 (**G**), CIRI2.0 (**H**) or KNIFE (**I**).

In the first simulation based on the data SRR444655 (Figure 3A–3C), CIRI1.2 respectively identified 2,762 and 2,111 circRNAs when using the first and second single-end reads, which was an apparent decrease compared to 3,488 circRNAs found by paired-end data SRR444655 (Figure 3A). A decrease in the number of predicted circRNAs was also observed when using CIRI2.0 and KNIFE algorithm (Figure 3B–3C). The number of true circRNAs for sing-end RNA-Seq data also decreased compared with paired-end data. Moreover, when only using the second single-end reads all prediction algorithms found much less circRNAs than using the first single-end reads (Figure 3A–3C). The number of both predicted circRNAs and true circRNAs predicted using the first single-end data exceeded one using the second single-end data, suggesting that the better reads quality (Supplementary Figure 1A) improve the predictions. Moreover, KNIFE algorithm almost did not work for single-end sequence.

In the second simulation (Figure 3D–3F), the number of predicted circRNAs decreased with the number of reads using for prediction linearly. A linear decrease was also observed in the number of true circRNAs for three algorithms. Meanwhile the true circRNAs was proportional to sequencing depth. For example, CIRI2.0 found about half of true circRNAs (461/1096) when sequencing depth was reduced to the half of SRR444655 (Figure 3E).

In the third simulation (Figure 3G–3H), the number of predicted circRNAs and true circRNAs decreased when more sequencing errors occurred in the reads. Our analysis showed that 794 true circRNAs were found by CIRI1.2 when 5 errors added in each read. Moreover, CIRI2.0 is more sensitive to sequencing error than CIRI1.2, having only 56 circRNAs predicted when 5 errors added and no circRNAs predicted when 10 errors added. KNIFE, being most sensitive to sequencing error, could not predict circRNAs when sequencing errors occurred in the reads. Comparing with the influence of single-end reads or sequencing depth on the prediction performance, sequencing error dramatically worsened the predictions.

To validate our findings we also employed CIRI2.0 and KNIFE algorithms to predict circRNAs from total RNA-Seq data SRR444975 and used three simulations to evaluate the prediction bias. The results on SRR444975 were consistent with our findings on SRR444655 (Supplementary Figure 3).

### Identifying the circRNA properties contributing to the efficient predictions

**Figure 4:**
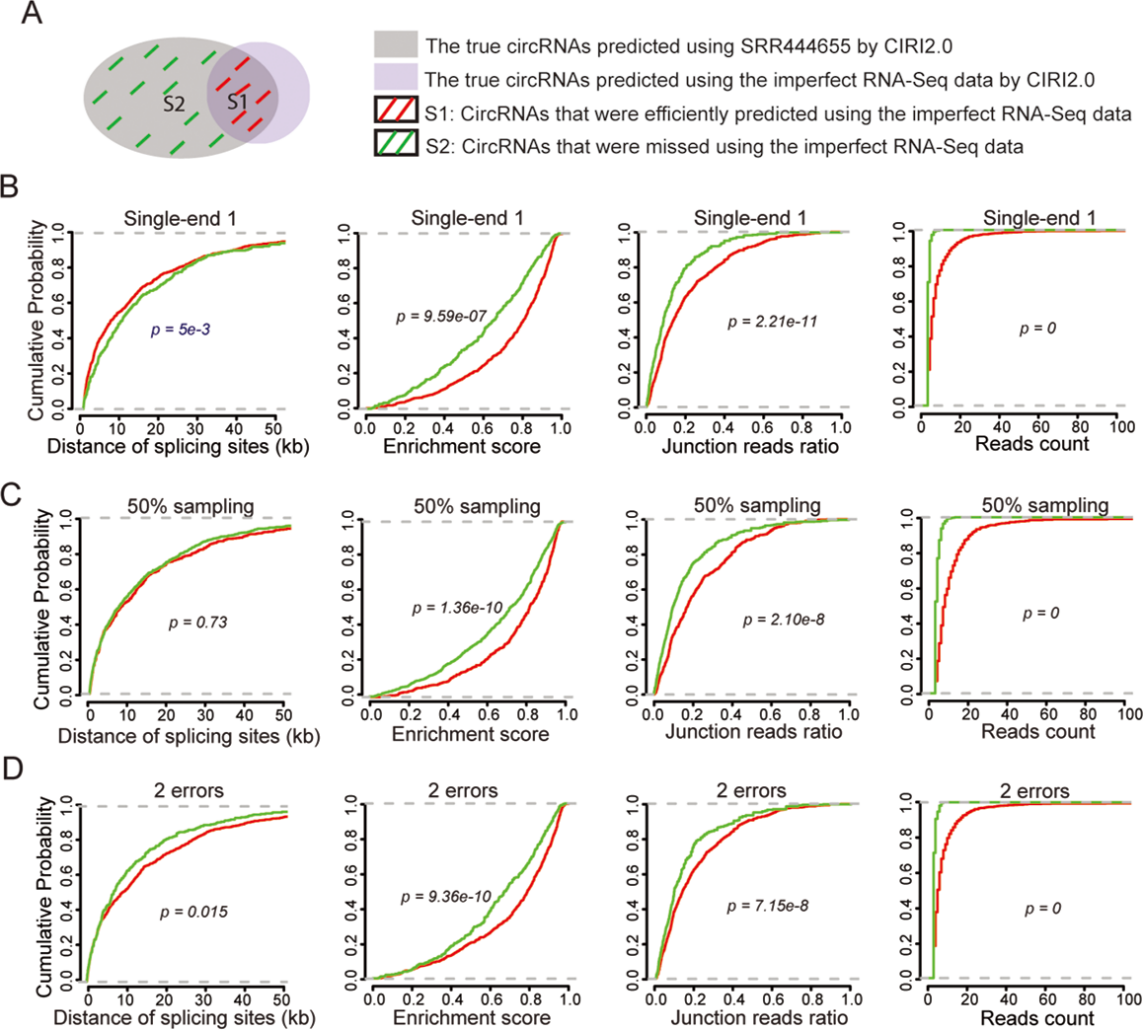
The circRNAs properties contributing to the efficient predictions using the imperfect total RNA-Seq data (**A**) Some cirRNAs were efficiently predicted by the imperfect RNA-Seq. Evaluation of the contributions of four types of circRNAs properties - distance of splicing sites, enrichment score, junction reads ratio, and reads count – to the efficient predictions using the first single-end RNA-Seq data (**B**) 50% sampling data (**C**) and data with the bases in each read were replaced by 5 false bases (**D**). Red curve is corresponding to cumulative distribution function (CDF) of the first group S1, and green curve is corresponding to cumulative distribution function (CDF) of the second group S2.

Our analysis above suggested that single-end reads, low sequencing depth and poor sequencing quality correlated with the decreased prediction performance. However, still a proportion of circRNAs was efficiently identified as true ones in the simulated imperfect RNA-Seq data. To identify the factors that may contribute to such efficient predictions, we evaluated four types of circRNAs properties - including distance between back-splicing sites, enrichment score, junction reads ratio, and reads count – between two groups of circRNAs (See methods). The first group (S1) covered the true circRNAs that were efficiently identified in the imperfect RNA-Seq data, and the second group (S2) covered true circRNAs that were not identified in the imperfect RNA-Seq data (Figure 4A). Kolmogorov-Smirnov test (K-S test) was employed to respectively compare cumulative distribution function (CDF) of these properties between two groups. Red curve is corresponding to CDF of the first group S1, and the green curve is corresponding to CDF of the second group S2 (Figure 4B–4D).

This evaluation was performed on three types of simulated imperfect data (single-end sequencing, low sequencing depth, and poor sequencing quality). The results showed that the shape of CDF for distance of splicing sites in S1 was similar to that in S2 (Figure 4B–4D, left panel), suggesting that distance of splicing sites had no difference between two groups. However, the shape of CDF for three types of properties - enrichment score, junction reads ratio, and reads count – in S1 was significantly right shifted in comparison with that in S2 (Figure 4B–4D, middle panels and right panels). This demonstrated that circRNAs with more reads count, higher junction reads ratio or greater enrichment score tend to be efficient predictable even in the imperfect RNA-Seq data. When we ranked the predicted circRNAs and selected the top 500 circRNAs with greater reads count, the true positives rate dramatically increased from around 50% to 70%. Therefore, though predictions from the imperfect total RNA-Seq data have a high risk to be artifacts, circRNAs with higher junction reads ratio and more reads count are much reliable. The evaluation when using other algorithm also showed the similar results (Supplementary Figure 4).

## DISCUSSION

In this study, we performed comparison analyses to systematically evaluate the bias of circRNA predictions from total RNA-Seq data. Our analysis showed that it was feasible to fully take advantage of the large amount of total RNA-Seq data in the public databases for circRNAs predictions. Though such prediction suffers from false positive, it can be carefully controlled by using total RNA-Seq data with good quality and preferentially selecting the circRNAs with high junction reads ratio and reads count.

Our analysis demonstrates that only total RNA-Seq data with good base quality is qualified for circRNA predictions. The number of true circRNAs decreased obviously along with the increasing sequencing errors (Figure 3G–3I), moreover, the second single-end RNA-Seq data with worse sequencing quality at 3′ termini gained less true positives (Figure 3A–3C). Therefore, sequencing error dramatically worsens the predictions, rather than single-end sequencing or low sequencing depth. However, though the ratio of false positive circRNAs was lower than that of true positive, among all of the circRNAs predicted from the perfect data, the absolute number of false positive circRNAs increased together with that of true positive ones. Therefore, there is still much space for further improvement of prediction performance for a single algorithm. And currently several algorithms could be combined to achieve reliable predictions.

In our study, we used CIRI1.2, CIRI2.0 and KNIFE to predict circRNAs. Different scircRNA prediction algorithms have different results. A recently published study [22] compared the predicted circRNAs from the different prediction algorithms. Their results suggested that combining two algorithms could effectively reduce the false positive, which was confirmed by our analysis. In addition, our analysis showed that CIRI2.0, as an updated version of CIRI1.2, gained the obviously improved true positives. KNIFE achieved the highest rate of true positive, and it worked efficiently on paired-end RNA sequencing data with good quality. But KNIFE greatly depended on sequencing strategy and sequence quality. Comparing with KNIFE, CIRI1.2 and CIRI2.0 still work on data with single-end RNA sequencing and poor sequence quality.

Our study has provided a comprehensive view on the predictions using the imperfect total RNA-Seq data. Our analysis demonstrates how public total RNA-Seq data with no sequencing costs, can be effectively utilized to promote the researches on circRNAs.

## MATERIALS AND METHODS

### Dataset analysis

RNA-Seq data of human fibroblast cell line Hs68, including total RNA sample (SRR444655, SRR444975) and RNase R treated sample (SRR444974, SRR445016) [2], was downloaded from the National Center for Biotechnology Information (NCBI) short Sequence Reads Archive. RNA sequencing data quality was investigated using FastQC (http://www.bioinformatics.babraham.ac.uk/projects/fastqc/). Using BWA-MEM alignment algorithm [24], we mapped the RNA-Seq reads to the human reference genome (hg19) that was downloaded from UCSC Genome Browser [25]. CIRI1.2, CIRI2.0 and KNIFE, the de novo circRNA identification algorithms, were employed to predict circRNAs with the default arguments. Only circRNAs with at least two reads spanning the splicing sites were kept for analysis. We designed an enrichment score to distinguish the true positives from false positives that were predicted from total RNA-Seq data. For a single predicted circRNA, enrichment score can be calculated by,

E=(m−n)/(m+n) (1)

Where *m* is the reads count of predicted circRNA from the Rnase R treated sample, and *n* is the reads count of predicted circRNA from total RNA-Seq sample. A predicted circRNA with enrichment score greater than 0 is considered as true positive.

### Prediction of simulated imperfect total RNA-Seq data

The imperfect RNA-Seq data were simulated based on total RNA-Seq data SRR444655 or SRR444975 in three ways: (i) Two files, the first single-end reads and the second single-end reads from SRR444655 or SRR444975, were simulated as two independent total RNA-Seq data with single-end sequencing. (ii) 80%, 50% and 20% reads were sampled from paired-end reads of SRR444655 or SRR444975, which simulated the gradually decreased sequencing depth. (iii) The bases in each read from SRR444655 or SRR444975 were replaced by other bases differing from them. For example, A is replaced by T, C, G, or N. 2, 5, 10 or 20 bases were substituted in each read, respectively, which simulated the gradually decreased sequencing quality. Simulated RNA-Seq data were carried out the same circRNA predictions above.

### Investigation of the circRNAs properties

Four types of circRNAs properties, including distance between back-splicing sites, enrichment score, junction reads ratio, and reads count, were investigated. Junction reads ratio can be calculated by,

$$R=\frac{n}{N}$$ (2)

Where *n* is the reads count of predicted circRNAs, and *N* is the reads count of predicted RNAs (including circRNA and linear RNA) around the circular region, a region between two junction sites.

Here true circRNAs were separated into two groups based on whether they were efficiently predicted in the imperfect RNA-Seq data. The first group covered the true circRNAs that were identified in the imperfect RNA-Seq data, and the second group covered ones that were not identified in the imperfect RNA-Seq data. Kolmogorov-Smirnov test (K-S test) was employed to respectively compare cumulative distribution function (CDF) of these properties between two groups of circRNAs.

## SUPPLEMENTARY MATERIALS FIGURES AND TABLES






