# Supplementary material for: Evaluating the bias of circRNA predictions from total RNA-Seq data
Source: Oncotarget. 2017 Dec 6;8(67):110914–21. doi: 10.18632/oncotarget.22972 (PMC5762294; doi:10.18632/oncotarget.22972)
Supplement: Supplementary file 1 [file oncotarget-08-110914-s001.pdf]

Evaluating the bias of circRNA predictions from total RNA-Seq data

SUPPLEMENTARY MATERIALS

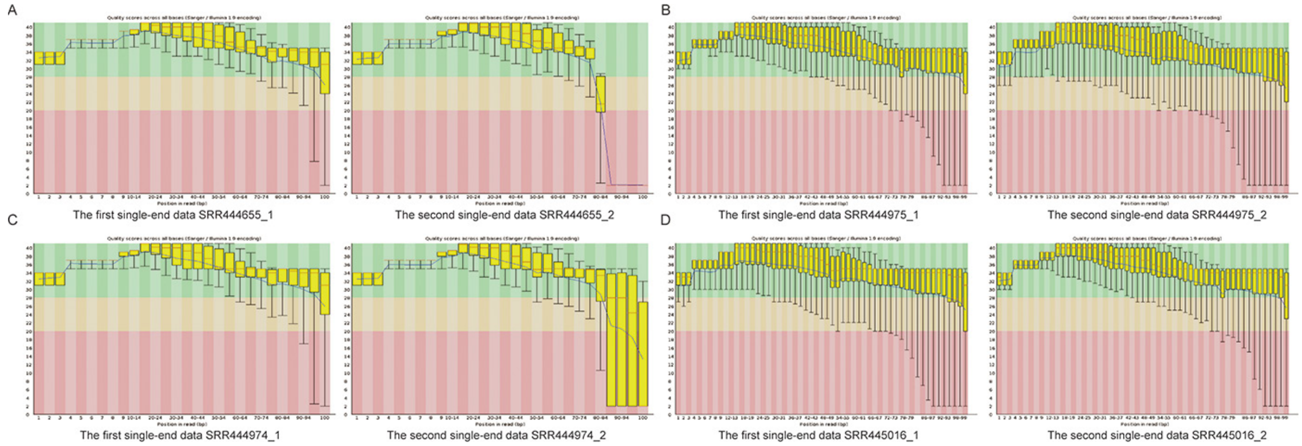

Supplementary Figure 1: FastQC evaluation of the data. (**A**) Quality score distributions across all bases of total RNA-Seq data SRR444655. (**B**) Quality score distributions across all bases of total RNA-Seq data SRR444975. (**C**) Quality score distributions across all bases of RNase R treated RNA-Seq data SRR444974. (**D**) Quality score distributions across all bases of RNase R treated RNA-Seq data SRR445016.

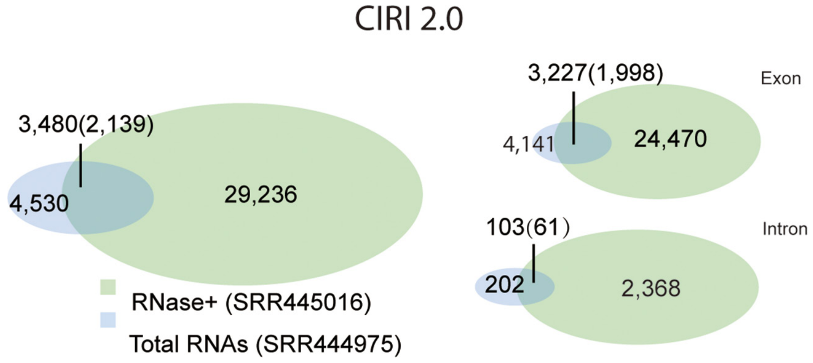

Supplementary Figure 2: Prediction performance of the total RNA-Seq data SRR444975 using CIRI2.0.

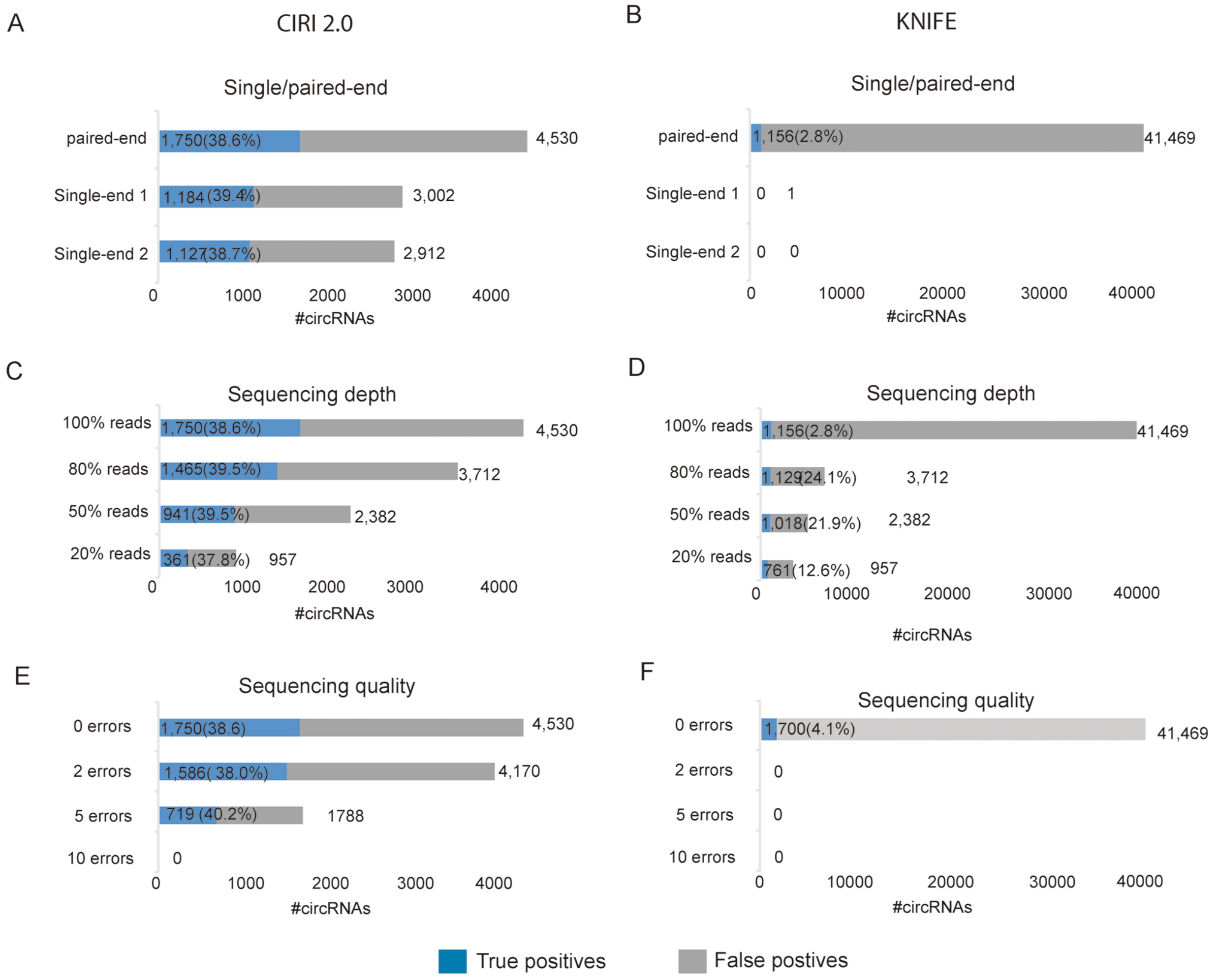

Supplementary Figure 3: Prediction performance of the imperfect total RNA-Seq data which were simulated based on total RNA-Seq data SRR444975. Prediction performance of the single-end sequencing data using CIRI2.0 (**A**) or KNIFE (**B**). Prediction performance of the data with gradually decreased sequencing depth using CIRI2.0 (**C**) or KNIFE (**D**). Prediction performance of the data with gradually increased sequencing error using CIRI2.0 (**E**) or KNIFE (**F**).

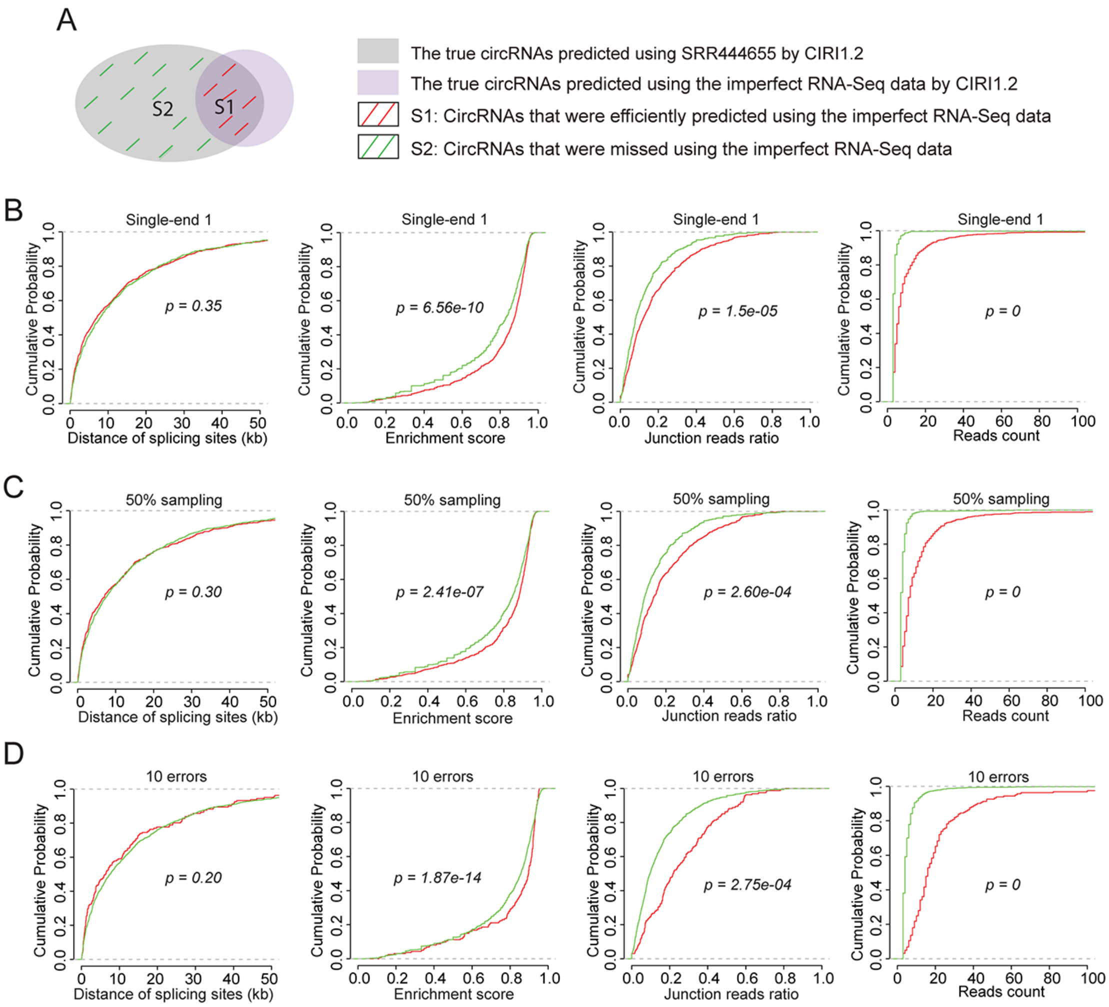

Supplementary Figure 4: The circRNAs properties contribute to the efficient predictions using the imperfect total RNA-Seq data (SRR444655, CIRI1.2).

Supplementary Table 1: The complete list of true circRNAs found using three algorithms. See Supplementary_Table_1

Supplementary Table 2: The complete list of false circRNAs found using three algorithms. See Supplementary_Table_2

Supplementary Table 3: The type of genes that generate circRNAs

	protein coding	lincRNA	pseudogene	other	total
CIRI1.2_True	1309	10	7	36	1362
CIRI2.0_True	1065	8	6	17	1096
KNIFE_True	1673	6	10	0	1689
CIRI1.2_Total	3067	22	27	372	3488
CIRI2.0_Total	2124	13	12	58	2207
KNIFE_Total	2777	8	15	54	2854
